# The Effect of Massage, Acupressure and Reflexology on Restless Legs Syndrome Severity and Sleep Quality in Patients Receiving Haemodialysis Treatment: A Systematic Review and Meta‐Analysis

**DOI:** 10.1002/nop2.70135

**Published:** 2025-02-04

**Authors:** Ayser Döner, Sultan Taşci, Aylin Bilgin

**Affiliations:** ^1^ Department of Internal Diseases Nursing, Faculty of Health Sciences Erciyes University Kayseri Turkey; ^2^ Department of Internal Medicine Nursing, Faculty of Health Sciences Sakarya University of Applied Sciences Sakarya Turkey

**Keywords:** acupressure, haemodialysis, massage, reflexology, Restless Leg Syndrome, sleep quality

## Abstract

**Aim:**

This study aimed to review massage, acupressure and reflexology interventions applied to haemodialysis patients with restless legs syndrome and to examine the effects of these interventions on sleep quality.

**Design:**

A systematic review and meta‐analysis.

**Data Sources:**

A systematic literature search was conducted on databases ScienceDirect, Web of Science, Cochrane Central Register of Controlled Trials, EBSCO and PubMed.

**Methods:**

The Modified Jadad scale was used. The statistical analysis was conducted using the Stata 15.0 software, with the aim of evaluating heterogeneity between studies using both chi‐square and *I* ^*2*^ statistics. The risk of bias in the included studies was assessed using both the Cochrane risk of bias assessment tool and statistical tests. A funnel plot was used to detect potential publication bias, which is indicated by an asymmetry in the plot. The Egger regression test was also performed to evaluate publication bias.

**Results:**

Twelve studies were selected for the meta‐analysis. All studies included in this meta‐analysis were of good quality. In the subgroup analysis, it was determined that massage and acupressure reduced the severity of restless legs syndrome as a result of the intervention, while reflexology had no effect. Interventions did not affect the sleep quality of patients. Restless legs syndrome severity was significantly reduced in studies using oil and in studies that intervened for more than 15 min per session.

**Conclusion:**

Interventions applied to haemodialysis patients with restless legs syndrome can reduce the severity of restless legs syndrome. It is recommended to conduct randomised controlled trials that determine the effects of methods on restless legs syndrome severity and sleep quality in haemodialysis patients.

## 1 Introduction

Although haemodialysis (HD) treatment, the most commonly used renal replacement therapy for end‐stage kidney disease (ESRD), increases the life expectancy of individuals, patients experience many physical and psychological symptoms such as pain, fatigue, restless legs syndrome (RLS), muscle cramps, insomnia, anxiety and depression (Bello et al. 2022; Rao et al. 2021). The prevalence of RLS, which is common in women and increases with age (Gossard et al. 2021), was found to be between 6.6% and 80% in HD patients (Gopaluni, Sherif, and Ahmadouk 2016). RLS is a common neurological sensorimotor disorder that usually occurs or worsens during periods of rest or inactivity, partially or completely relieved by movement. The main symptom in RLS is the urge to move the legs because of unpleasant or uncomfortable sensations in the legs. Although the name of the symptom is RLS, as the severity of RLS increases, symptoms can also be seen in other parts of the body such as arms, trunk and hips. However, the legs are affected more severely than other parts of the body (Gossard et al. 2021; Vlasie et al. 2022). Patients on HD treatment often suffer from RLS due to the long sedentary time in the HD session (Xia et al. 2022). RLS symptoms may negatively affect patients' participation in HD by preventing them from remaining sedentary during HD (Chaiard and Weaver 2019). RLS may be accompanied by symptoms such as pain and cramps, or these symptoms may increase the severity of RLS (Döner and Taşcı 2022b). Studies have shown that RLS symptoms increase the level of fatigue (Giannaki et al. 2017), daytime sleepiness (Zhang et al. 2020) and use of sleeping pills (Lin et al. 2019), and adversely affect sleep quality (SQ) (Giannaki et al. 2017; Lin et al. 2019; Zhang et al. 2020) and QoL (Kutlu et al. 2018).

Complementary and integrative treatment (CIT) and pharmacological methods are frequently used in the management of sleep disorders and RLS symptoms experienced by HD patients (Chen et al. 2022; Kesik and Ersoy 2023). Drugs used in pharmacological treatment can cause serious side effects. It has been determined that some drugs cause the development of RLS or worsen RLS symptoms. For this reason, HD patients prefer CIT methods (Chaiard and Weaver 2019; Vlasie et al. 2022). CIT methods used to provide RLS symptom management focus on individuality and strengthen the patient in the healing process with a holistic perspective (Döner and Taşcı 2022b). It is seen that interventions such as intradialytic exercise, aromatherapy, massage therapy, reflexology and acupressure, which are among the CIT methods, are frequently used in the management of sleep disorders and RLS (Chen et al. 2022; Harrison et al. 2019; Huang et al. 2021; Xu et al. 2018). Massage therapy, which is one of the frequently used CIT methods, has been used for a long time in most cultures to relieve and alleviate many ailments. There are many different types of massage therapy, which is also defined as mechanical or manual stimulation of the soft tissues of the body. The most commonly used type is classical massage, and in this massage, effleurage, petrissage, friction, tapotman and vibration techniques are used (Salvo, Sefton, and Corpus 2019). In massage therapy, essential oils such as lavender and sweet orange oil, which are generally used in aromatherapy, base oils such as olive oil and sweet almond oil or oils such as liquid vaseline, glycerine and baby oil are used as lubricants (Döner and Taşcı 2022a, 2022b; Manion and Widder 2017). Essential oils used in aromatherapy massage are easily absorbed by the skin, and have some therapeutic effects (Babar et al. 2015). Acupuncture is applied with needles to stimulate certain anatomical points on the meridians in the body to restore the body's normal function and energy balance. Manual pressure (acupressure), electrical stimulation (electroacupuncture) and low‐power lasers (laser acupuncture), heat and ultrasound are also used to stimulate these points in the acupuncture treatment method (White, Cummings, and Filshie 2018). Acupressure is done by applying pressure to specific acupuncture points with hands, thumbs or a stimulation device to activate the body's natural self‐healing process. Finger and palm acupressure is classified as a subtype of therapeutic massage (Mehta et al. 2017). Reflexology, on the other hand, is the intervention of manual pressure to reflex points in the hands, ears and feet associated with organs and body parts. Reflexology is applied to balance the energy in the body, to provide healing and relaxation (Döner and Taşcı 2022b). Aghajani, Kheirkhah, and Hashemi (2016) found that effleurage massage with glycerine oil reduced the severity of RLS symptoms. Li et al. (2021) reported that electroacupuncture applied to acupuncture points in the lower extremity reduced the severity of RLS symptoms and improves the SQ. Determined that reflexology reduced the severity of RLS. As a result, massage therapy and acupressure interventions have many positive effects on HD patients with RLS. A holistic perspective will be developed when massage therapy and acupressure intervention duration, intervention protocol and patient inclusion criteria are determined based on evidence. This systematic review and meta‐analysis provide evidence for a holistic assessment and synthesis of the effects of massage and acupressure on RLS symptom management and the SQ in HD patients with RLS. This systematic review and meta‐analysis aimed the following: (i) to reveal the effects of massage therapy and acupressure on RLS symptom management in HD patients, (ii) to reveal the effects of massage therapy and acupressure on SQ, (iii) to learn about the methodological quality and level of evidence of the studies included in this meta‐analysis and (iv) to provide evidence‐based recommendations for massage therapy and acupressure interventions.

## 2 Methods

### 2.1 Study Design

This study was conducted appropriately ‘the Preferred Reporting Items for Systematic Reviews and Meta‐Analysis (PRISMA) checklist’ (Page et al. 2021) (Data S1). The meta‐analysis was guided by a five‐dimensional PICOS strategy, which included the elements such as ‘Participants (P), Intervention (I), Control (C), Outcome (O) and Study design (S)’ (Schardt et al. 2007). The participants (P) included in the meta‐analysis were individuals undergoing HD treatment, while the interventions (I) studied were massage, acupressureand reflexology administered to these patients. The control (C) group consisted of patients who had not received any of these interventions. The outcome (O) measures of interest were RLS severity and SQ. Finally, only randomised controlled trials (S) were included in the meta‐analysis. Thus, the research question for the current meta‐analysis was created as follows: ‘What is the impact of massage, acupressure, and reflexology on RLS severity and the SQ in HD patients?’

### 2.2 Eligibility Criteria

This meta‐analysis involved articles that were (a) performed on patients undergoing HD treatment, (b) applied massage, acupressure and reflexology, (c) reported the RLS severity and SQ as assessment outcome, (d) were written in English and (e) had a randomised controlled design. Studies that only provided abstracts or did not include both standard deviation (SD) and mean (M) values necessary to determine the effect size were not involved in this meta‐analysis. Additionally, this meta‐analysis were excluded grey literature sources, such as dissertations, letters, expert opinions, conference papers and non‐peer‐reviewed journal articles. Moreover, patients receiving peritoneal dialysis treatment were excluded from the study.

### 2.3 Search Strategy

To identify relevant studies, a systematic literature search was conducted on multiple databases including “Cochrane Central Register of Controlled Trials (CENTRAL)”, “ScienceDirect”, “Web of Science”, “EBSCO” and “PubMed”. The search was not limited by publication date. PICO headings were used to define search terms for the population, intervention, comparator and outcome. The search terms included variations of (1) “Restless leg syndrome” OR “Restless Legs Syndrome” OR “Willis‐Ekbom disease” OR “RLS” OR “WED” OR “uremic RLS” OR “secondary RLS” AND (2) “Hemodialysis” AND (3) “Aromatherapy” OR “Aroma” OR “Massage” OR “Reflexology” OR “Acupressure” OR “Acupuncture” OR “Acupoint” (Data S2). Two authors independently screened the studies for inclusion, and all relevant studies were imported into the EndNote Citation Software X20 (Clarivate Analytics, Philadelphia, USA). The systematic literature search was conducted to ensure a comprehensive and unbiased selection of studies for the meta‐analysis, using a standardised and replicable search strategy.

### 2.4 Data Extraction

After importing all the studies into the Endnote X20, the authors reviewed them to remove duplicates. Subsequently, the studies were screened based on their titles and abstracts by two researchers who assessed them against the exclusion and inclusion criteria. Subsequently, the full‐text versions of the selected studies were independently evaluated by the researchers according to the exclusion and inclusion criteria. To standardise the evaluation process, the researchers created a form that included various study characteristics such as the first author, publication date, country, HD treatment time, RLS criteria, gender, age, sample size, intervention strategies, duration of interventions, massage oil information, training providers, control strategies, scales and assessment times. Inconsistencies in the data achieved from the articles were resolved with discussion among the authors.

### 2.5 Risk of Bias and Quality Appraisal Assessment

Modified Jadad scale was used for evaluate the methodological quality (Oremus et al. 2001). The scale includes eight items related to withdrawal and dropout rate, exclusion and inclusion criteria, randomisation, blinding, adverse effects, procedures and statistical tests. Each item is scored as ‘yes’ or ‘no’, with ‘no’ scored as ‘0’ and ‘yes’ scored as ‘1’. The total score for each study is obtained by summing the scores for each item, ranging from 0 (low) to 8 (high). A quality score of 3 or below were considered to be of ‘low quality’, while a score of 4 or above were considered to be of ‘good quality’. Two researchers independently assessed the methodological quality of each study, and any inconsistencies were resolved through discussion and consensus.

The Cochrane risk of bias assessment tool were used to assess the risk of bias in the included studies (Corbett, Higgins, and Woolacott 2014). The Cochrane tool assesses six types of bias, including performance, selection, detection, reporting, attrition and other biases. Each type of bias is rated as high, unclear or low risk. A funnel plot was utilised to detect potential publication bias, which is indicated by an asymmetry in the plot. Also, publication bias was evaluated with the Egger regression test. Three researchers independently assessed the risk of bias of each study, and any discrepancies were resolved with discussion.

### 2.6 Data Analysis

The statistical analysis was conducted using Stata 15.0 software to evaluate heterogeneity between studies using both chi‐square and *I* ^2^ statistics. The *I* ^2^ values were categorised into four groups: high heterogeneity (75%–100%), moderate heterogeneity (50%–75%), low heterogeneity (25%–50%) and no heterogeneity (0%–25%). A random effects model was utilised for the meta‐analysis, and studies with an *I* ^2^ > 50% and a *p* value < 0.5 were included. The results were standardised using both 95% confidence interval (*CI*) and standardised mean difference (SMD), which allowed for direct comparison of results on the same scale. The forest plot was used to visually represent the analysis data. Additionally, meta‐regression and subgroup analysis were used to determine the impact of specific study characteristics and variables, such as intervention, HD treatment time, duration of intervention, session duration and massage oil.

## 3 Results

### 3.1 Summary of Search Outcomes

**FIGURE 1:**
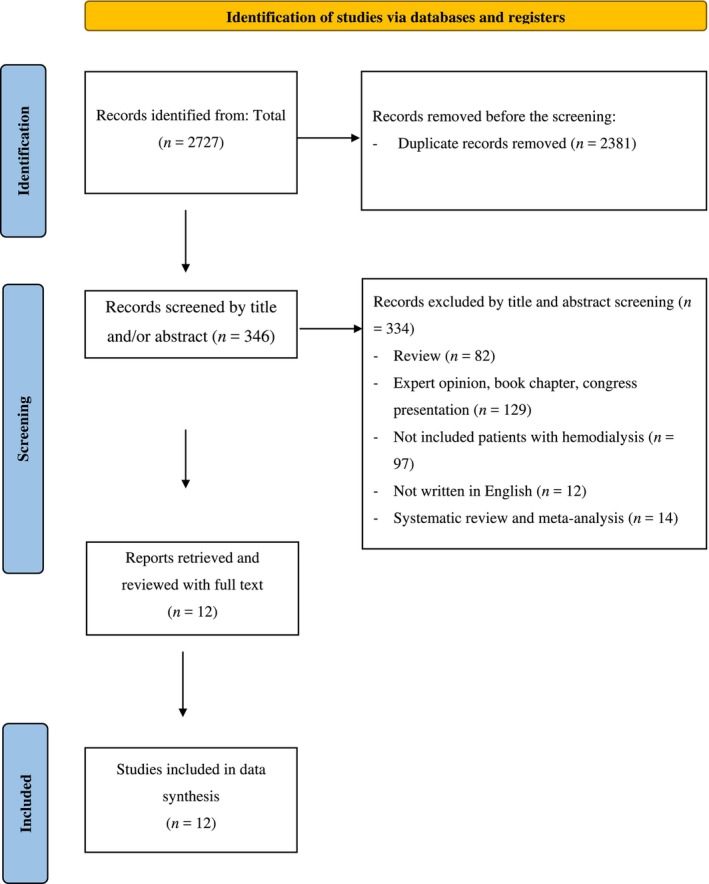
PRISMA flow diagram (Page et al. 2021).

Four databases were queried, yielding 2727 studies. After excluding 2381 repetitive studies, the remaining pool underwent initial screening based on titles and abstracts, resulting in the exclusion of 334 studies that did not meet the criteria. Twelve studies underwent full‐text review, leading to the inclusion of these in the meta‐analysis (Figure 1).

### 3.2 Characteristics of Studies

The majority of the studies (*n* = 10) were conducted in Iran. One of the remaining studies was conducted in Taiwan and one study was conducted in Turkey. In seven of the included studies, patients had been receiving HD for at least 3 months, in four for 6 months and in one for 1 year. In the majority of the studies, the presence of RLS was evaluated according to the International RLS Study Group diagnostic criteria. The sample size in the studies varied between 23 (Tsai et al. 2022) and 80 (Azimpour et al. 2019). The lowest mean age was 48.17 ± 1.65 years and the highest mean age was 64.96 ± 11.09 years. In almost all the studies, both male and female patients were included; however, all participants were female in one study (Ghasemi et al. 2021) (Table 1). When the intervention strategies in the studies were analysed, massage therapy was used in eight studies, reflexology in two studies and acupressure in two studies. Eight of the studies provided information about the oils used during the intervention. Lavender oil was mostly used in the studies. The interventions lasted at least 3 weeks and at most 8 weeks. Each session lasted at least 10 and at most 45 min. Nine of the studies was emphasised that intervention was implemented by trained, experienced or certified health professionals (Table 2).

**TABLE 1:** Characteristics of reviewed studies.

Study (years)	Country	Inclusion criteria	Haemodialysis treatment time	RLS criteria	Sample	Age (mean ± SD)	Gender (per cent)
Amrollahi et al. (2022)	Iran, Yazd	Chronic renal failure necessitating dialysis Having the ability to communicate verbally Having the physical and mental ability Ferritin > 100 ng/mL Transferrin saturation > 20% Lack of wounds and redness of the limbs No allergic reaction to lavender A healthy olfaction (sense of smell) No history of asthma, migraines and/or allergies	At least 3 months with three sessions per week	RLS according to IRLSSG diagnostic criteria (all four diagnostic criteria) Patients were examined for motor and neurologic examination by a nurse to ensure they were not impaired by nerve and peripheral neuropathy	Intervention group = 20 Control group = 21	Intervention group = 55.10 ± 15.37 Control group = 57.12 ± 13.67	Intervention: Female = 40.0% Male = 60.0% Control: Female = 47.60% Male = 52.40%
Azimpour et al. (2019)	Iran, Meibod, Ardakan and Yazd	Lack of consciousness The absence of any malignancy, ulcer and dermal erythematosus Not using vibration and massage at home.	At least 3 months after haemodialysis	Presence of RLS (11+ points)	Intervention group = 40 Control group = 40	Two groups = 61.41 years	Female = 42.5% Male = 57.5%
Döner and Taşcı (2022a)	Turkey, Kayseri	Age ≥ 18 years Not pregnant or planning pregnancy Diagnosed with RLS by a physician Had at least mild RLS according to the RLS severity rating scale Not changed the treatment schedule before and throughout the study No allergy to lavender oil Not having an open wound that would hinder massage therapy No lower leg problems such as neuropathy No neurological diseases, such as Parkinson's disease, Alzheimer's disease, epilepsy or multiple sclerosis	At least 3 months with three sessions per week	The researcher identified patients diagnosed with RLS by a physician	Intervention group = 31 Control group = 27	Intervention group = 63.19 ± 10.57 Placebo control group = 64.96 ± 11.09	Intervention: Female = 54.8% Male = 45.2% Control: Female = 44.4% Male = 55.6%
Ghanbari et al. (2022)	Southeastern Iran	Being 18–65 years old Having no medical prohibition for the intervention such as foot ulcers, amputation, and orthopaedic problems Having no debilitating and chronic diseases such as cancer, chronic respiratory failure, heart failure, rheumatoid arthritis and lupus erythematosus Having no psychological disorders known as depression and bipolar disorder according to the patient's self‐declaration	At least 3 months with three sessions per week and lasting 4 h each time	RLS according to IRLSSG diagnostic criteria (all four diagnostic criteria)	Intervention group = 30 Control group = 30	Intervention group = 51.66 ± 5.42 Placebo group = 51.56 ± 5.50	Intervention: Female = 43.3% Male = 56.7% Control: Female = 43.3% Male = 56.7%
Ghasemi et al. (2021)	Iran, Tehran	Female gender Age above 18 years Not taking anxiolytics and sedative medications for at least last 4 h before the interventions Having no history of alternative and complementary care in the last 48 h The absence of foot ulcers Having no history of drug addiction, asthma, eczema and allergy No history of mental or physical disability including vascular problems in legs or peripheral neuropathy	Three times a week for 6 months and each session lasting for 3–4 h	Diagnosis of RLS by a physician, and RLS according to IRLSSG diagnostic criteria (all four diagnostic criteria) Patients diagnosed with RLS by a physician	Intervention group = 35 Control group = 35	Intervention group = 48.17 ± 1.65 Placebo group = 50.45 ± 1.13	Female = 100%
Hashemi, Hajbagheri, and Aghajani (2015)	Iran, Kashan and Qom	Age 18–65 years Having a medical diagnosis of chronic renal failure Not having a history of allergy to plants Having no active wound below the knee Not having a known psychiatric disorder, dementia and mental retardation Being able to walk on his or her feet	Three times a week for 6 months	—	Intervention group = 29 Control group = 30	Intervention group = 57.5 ± 14.6 Placebo group = 56.10 ± 13.56	Intervention: Female = 62.1% Male = 37.9% Control: Female = 43.3% Male = 56.7%
Ajorpaz et al. (2020)	Iran, Kashan and Qom	Ages between 18 and 65 years Having a medical diagnosis of CRF Able to walk without support No history of allergy No active wound below the knees No substance addiction, additional physiological or physical disorders, such as muscular dystrophy or known psychiatric or cognitive disorders No having history of deep vein thrombosis (DVT) Not receiving painkillers in the 3 hours prior to intervention	Three or four times a week for 6 months	—	Intervention group = 29 Control group = 30	Intervention group = 57.5 ± 14.6 Placebo group = 56.10 ± 13.56	Intervention: Female = 18 Male = 11 Control: Female = 13 Male = 17
Mohammadi et al. (2018)	Iran, Kermanshah	Between the ages of 18 years and 65 years No vascular access in the leg area, such as an arteriovenous shunt The absence of peripheral neuropathy and vascular problems in the lower extremities Not pregnant Not currently taking drugs for RLS, such as dopamine agonists, benzodiazepines, opioids and gabapentin No history of other motor disorders, such as Parkinson's disease, dyskinesia and dystonia	At least 3 months with three sessions per week	RLS according to IRLSSG diagnostic criteria (all four diagnostic criteria)	Intervention group = 30 Control group = 30	Intervention group = 56.11 ± 12.66 Control group = 57.36 ± 12.14	Intervention group: Female = 40.0% Male = 60.0% Control group: Female = 46.7% Male = 53.3%
Nasiri et al. (2019)	Iran, Qom	Age range of 18–65 years Being fully conscious Having the ability to walk independently with the affected feet Experience of RLS symptoms at least twice a week No sensitivity or allergy to the herbal extracts No using any herbal extracts (topical or oral) in the past 3 months No presence of any wound, fracture, amputation and trauma below the knee Not having a history of addiction to drugs or alcohol Not having any mental disorders, dementia or intellectual disability	At least twice per week for 6 months and each session lasting for 3–4 h	The diagnostic criteria were assessed by a nurse under the supervision of a nephrologist RLS according to IRLSSG diagnostic criteria (all four diagnostic criteria) The patients were assessed for mimic conditions by a neurologist and orthopaedics, and those with these conditions were not included in this study	Intervention group = 27 Control group = 28	Olive oil group = 50.55 ± 11.07 Placebo group = 53.71 ± 11.55	Olive oil group: Female = 55.6% Male = 44.4% Placebo group: Female = 42.9% Male = 57.1%
Oshvandi et al. (2021)	Iran, Hamadan	An age range of 30–75 years Healthy olfactory function and no history of allergic rhinitis or respiratory disorders No allergy to aromatic herbs No participation in aromatherapy or massage therapy programs during the last 6 months before the study Not to take any sleeping pill before aromatherapy and during the study No history of foot amputation or active skin lesion in the feet No addiction to opioids No affliction by debilitating chronic physical conditions such as cardiac, respiratory, liver or mental disorders according to patients' medical records	At least 1 year with three sessions per week	RLS according to IRLSSG diagnostic criteria (all four diagnostic criteria)	Intervention group = 35 Control group = 35	Three groups = 51.87 ± 8.73	—
Shahgholian et al. (2016)	Iran, Isfahan	Aged 18–65 years Haemodialysed with bicarbonate solution had no idiopathic RLS Not consuming medications to manage RLS signs or medications worsening these signs (three‐cycle antidepressants, serotonin selective reuptake inhibitors, anti‐nausea medications, antiepileptics, antipsychotics and dopamine antagonists) No infection, wound or serious complication in feet, and peripheral neuropathy or vascular problems in lower limbs	Three times a week for 3 months and each session lasting for 4 h	RLS according to IRLSSG diagnostic criteria (all four diagnostic criteria)	Intervention group = 30 Control group = 30	Three groups = 55.45 ± 12.08	Female = 50.0% Male = 50.0%
Tsai et al. (2022)	Northern Taiwan	At least 20 years old Clear consciousness and ability to communicate in Mandarin or Taiwanese No history of malignancy Intact skin and no impairment in the lower extremities Stable vital signs and no bleeding tendency	At least 3 months	RLS according to IRLSSG diagnostic criteria (all four diagnostic criteria)	Intervention group = 14 Control group = 9	Intervention group = 35–64 years = 71.43% ≧ 65 = 28.57% Control group = 35–64 years = 77.78% ≧ 65 = 22.22%	Intervention group = Female = 35.71% Male = 64.29% Control group = Female = 22.22% Male = 77.78%

Abbreviations: HD = haemodialysis, IRLSRS = International Restless Legs Syndrome Rating Scale, IRLSSG = the International Restless Legs Syndrome Study Group, RLS = Restless Legs Syndrome, SD = standard deviation.

**TABLE 2:** Intervention strategies and results of reviewed studies.

Study (years)	Intervention	Duration of intervention	Massage oil information	Intervention strategies	Control strategies	The training providers	Scales	Assessment times
Amrollahi et al. (2022)	Massage	Each session lasting 30 min, three times a week for four consecutive weeks	Lavender oil was used by Barij Essence Pharmaceutical Company. Each massage therapy session utilised 10 cc of lavender oil	The intervention group received 30‐min sessions of effleurage massage with lavender oil on Sundays, Tuesdays and Thursdays during the second to third hours of dialysis. The effleurage technique, which involves gentle whole‐hand movements in the direction of blood flow from the wrists to the knees, was used on the extremities	Routine interventions were performed and they were only asked to move their legs during HD to relieve symptoms	The massage was performed by same‐sex nurses, colleagues of the patient who had been trained	IRLSSG RLS rating scale for severity	Before 1 week after the intervention
Azimpour et al. (2019)	Massage	Each session lasting 10 min, three times a week for four consecutive weeks	—	Massage therapy was administered to the patients during dialysis sessions for 1 month. The massages were conducted by a trained therapist three times a week, with each session lasting 10 min. The technique employed for the massages involved the use of hacking massage on both feet, which uses rapid sequential hacking motions without any pauses, performed with the side of the hands using two modalities	The patients underwent vibration therapy for one month on both feet at the time of haemodialysis, and vibration was conducted at low voltage	Massaging was performed by a trained person	IRLSSG RLS rating scale for severity The Pittsburgh Sleep Quality Index (PSQI)	Before 4 weeks
Döner and Taşcı (2022a)	Massage	Each session lasting 20 min, three times a week for four consecutive weeks	Expert recommendation was followed to prepare a 5% mixture of 100 mL sesame oil and 5 mL lavender essential oil. The lavender oil (Lot/Batch No: 0144) was obtained from Sarl Pyrenessences Analyses, Belcaire, France, while the sesame oil (Batch No: 008901) was obtained from Helvacızade Food Pharma & Chemicals Inc. Konya, Turkey	During the massage, the windows in the HD booth were kept open without the use of air conditioning or ventilation devices. After each HD session, the room was ventilated for at least 1 hour by opening all the windows before the next session. To ensure consistency in the massage treatment, a massage protocol was developed for the interviewers who would also serve as massage therapists, outlining the massage steps and important points to consider	Baby oil was used for the patients in the control group	The study involved patients from seven different centres, and four interviewers were selected to serve as massage therapists. The interviewers, who were recent graduates of a 4‐year nursing program and had no work experience, received three hours of theoretical and practical training on massage techniques from the researchers	IRLSSG RLS rating scale for severity Kidney disease quality of life short form	Before 1 week 2 weeks 3 weeks 4 weeks
Ghanbari et al. (2022)	Reflexology	20 min on both feet, three times a week for four consecutive weeks	The researcher used 2–3 cc of baby oil to soften the skin and ease the massage so as not to irritate the patient	The masseurs started by supporting the toes with one hand and massaging the soles of the feet from top to bottom using the second finger of their other hand. They applied gentle pressure to the inner edge of the ankle while massaging the tip of their hand from the big toe. They massaged the reflex points from the heel of one foot to the thumb of the opposite hand using alternating and reciprocal pressure. Using the palm of their hand, they moved the outer edge of the patient's foot back and forth while using gentle, fast‐paced movements to massage relevant reflex points on the outside of the big toe. Finally, the masseur provided a 1‐min general massage	A simple touch was applied to the patients from the knee to the sole of the foot without pressing the reflexology points in the same condition and time	—	IRLSSG RLS rating scale for severity PSQI	Before 4 weeks 1 month (for follow‐up) after the intervention
Ghasemi et al. (2021)	Massage	15 min on each foot, three times a week for eight consecutive weeks	The lavender essential oil was selected with guidance from pharmacology experts at the university. The oil was comprised of linalool (27.11%) and linalyl acetate (23.33%) in a 3:3:2:2 mL ratio per 100 mL of coconut carrier oil	Aromatherapy massage using lavender essential oil was applied to induce relaxation. The therapist applied gentle pressure with their hands on the patient's foot and also used their fist to apply pressure, adjusting the intensity based on the patient's comfort level. Additionally, the therapist massaged the same reflex points on the foot using 10 drops of lavender essential oil to enhance the therapeutic effect of the massage	The same therapist massaged the reflex points of the feet using almond oil for similar durations as the intervention groups, without stimulating the reflex points	The female researcher who had received education about foot reflexology performed the interventions	IRLSSG RLS rating scale for severity	Before 4 and 8 weeks of the interventions
Hashemi, Hajbagheri, and Aghajani (2015)	Massage	Each session lasted 10 min, twice times a week for three consecutive weeks	A 1.5% concentration of lavender oil extracted from unopened lavender blossoms (10–15 mL) was used for the massage, which was prepared by Barij Essence Pharmaceutical Company in Kashan, Iran	A lavender oil‐based simple effleurage massage was administered to both legs, from the sole of the foot to the knee. The massage began with thumb movements from the toes towards the heel of the foot, followed by deep palm and thumb pressure on the back of the leg from the ankle to the knee, and then back down to the foot with a light pressure. The massage was timed according to the patients' routine haemodialysis sessions	Patients in the control group received their routine haemodialysis without any classic massage or aromatherapy	The trained researcher nurse performed the intervention for males and a trained female assistant did the intervention for females	IRLSSG RLS rating scale for severity	Before 1 week after the intervention
Ajorpaz et al. (2020)	Massage	Each session lasted 45 min, three times a week for four consecutive weeks	The concentration of lavender oil used for the massage was determined after consulting with an herbal medicine specialist and considering relevant studies. The massage therapist used 10–15 mL of lavender oil with a 1.5% concentration	The effleurage massage, a technique using long, light or firm strokes, was used in massage therapy. This technique is known to promote circulatory and immune systems, skin and muscle tone and relaxation. Initially, effleurage massage was applied to each leg, starting from the plantar surface of the toes towards the heel using thumb movements. Following this, the back of the calves was massaged with deep pressure applied using the palms of the hands and thumbs, from the ankle up to the knee and back down to the foot	Patients received no additional treatment	Male participants received interventions from a certified male nurse, while interventions for female participants were provided by a certified female nurse	IRLSSG RLS rating scale for severity	Before 1 week after the intervention
Mohammadi et al. (2018)	Acupressure	Each session lasted 10 min, three times a week for four consecutive weeks	—	The BioBeam 940 light source was used to administer NIR light therapy sessions to the participants. The plantar surface of each foot was exposed to NIR light with a radiation angle of 90°, and each acupoint received energy transfer for 2 min. The ‘bathing method’ was used to transfer the energy to the foot by moving the infrared probe across the plantar surface for 2 min. Zusanli (ST36), Sanyinjiao (SP6), Yang Ling Quan (GB34), and Cheng Shan (BL57) points were the acupoints that received the NIR light. The concentration of energy in each acupoint was monitored to ensure consistency	The participants received a sham treatment, where the researcher performed the same procedure as in the intervention group but without turning on the NIR light therapy device	A trained physical therapist performed the intervention	IRLSSG RLS rating scale for severity	Before 2 weeks 4 weeks 6 weeks
Nasiri et al. (2019)	Massage	Each session lasted 10 min, twice times a week for three consecutive weeks	The olive oil used in the study was a refined, pure and odourless type obtained from Loyeh Ind., Gilan, Iran. The olive oil had a health production licence No. of 47/10794 and a production serial No. of PZS1626815. The placebo used in the study was an oral lemonade liquid paraffin obtained from Farabi Pharmaceutical & Cosmetic Co., Tehran, Iran	Before each haemodialysis session, the patients washed their legs with 1000 mL of normal saline solution (0.9%). In the olive oil group, patients received a 10 mL application of olive oil to one leg, from the plantar surface of the foot to the area below the knee, and a 5‐minute massage using the palm of the hand or fingers with light pressure stroking technique. The massage began from the plantar surface of the foot towards the heel and the dorsal surface of the foot towards the ankle. Then, the posterior and anterior sides of the leg were massaged up to the knee. The same technique was applied to the other leg	The placebo group received massage with liquid paraffin twice a week during HD sessions for 3 weeks	Two nurses were trained on how to apply the massage in a formal session by the main researcher, who had experience in massage therapy and the application of herbal oils	IRLSSG RLS rating scale for severity	Before 1 week after the intervention
Oshvandi et al. (2021)	Massage	Each session lasted 18 min, three times a week for three consecutive weeks	In this study, 10–15 cc of essential oil 1.5% from each one (lavender and sweet orange) were (purchased from Barij Essence Co. Ltd., Kashan, Iran)	The effleurage massage technique was used in this study. The patient was placed in a supine position on a comfortable bed, and the massage was performed 1 h after the haemodialysis session. The massage started at the inner surface of the foot and progressed towards the knee, followed by a return stroke from the knee to the foot. The shins were massaged using two or three fingers from the knee to the foot. The hands were moved from the inner surface of the shins up and down on the back of the gastrocnemius and soleus muscles. The hands were moved from the ankle to the toes and then down from the side of the metatarsal area	The control group received routine care, and massage without essential oil	Male participants received interventions from a male researcher, while interventions for female participants were provided by a female researcher	IRLSSG RLS rating scale for severity PSQI	Before After the intervention At the end of the first week, the second and third weeks
Shahgholian et al. (2016)	Reflexology	Each session lasted 30–40 min, three times a week for four consecutive weeks	—	Interventions of reflexology were administrated for two groups as three sessions a week (12 sessions), each session lasting for 30–40 min in the first 2 h of dialysis session in which there were no notable changes in blood pressure respectively	The control group received the usual care	—	IRLSSG RLS rating scale for severity	Before 4 weeks
Tsai et al. (2022)	Acupressure	Each session lasted 36 min, three times a week for four consecutive weeks	—	12 specific acupoints on the legs were chosen for acupressure treatment, which included Zusanli (ST36), Yanglingquan (GB34), Sanyinjiao (SP6), Xuanzhong (GB39), Chengshan (BL57) and Taichong (LR3). The acupressure was performed using fingertip rolling at a constant rate of about two or three rollings per second, and each acupoint was pressed for 5 s, followed by a 1‐s relief period and then repeated for a total of 3 min	The control group received the usual care	The trained clinical nurse performed the intervention	IRLSSG RLS rating scale for severity PSQI	Before First month Second month

Abbreviations: HD = Haemodialysis, IRLSRS = International Restless Legs Syndrome Rating Scale, IRLSSG = the International Restless Legs Syndrome Study Group, PSQI = the Pittsburgh Sleep Quality Index, RLS = restless legs syndrome.

### 3.3 Quality Appraisal

The randomisation method was used in all studies. Blinding was used in some studies, double‐blind in one study, and single‐blind in five studies. Moreover, all studies meticulously outlined their inclusion/exclusion criteria, withdrawal rates and employed fitting statistical analyses. Notably, only seven studies documented adverse effects of the intervention, with none reporting any side effects. When considering the total scores, five studies (Amrollahi et al. 2022; Ghasemi et al. 2021; Hashemi, Hajbagheri, and Aghajani 2015; Mohammadi et al. 2018; Nasiri et al. 2019) scored 8 points, one study (Ajorpaz et al. 2020) scored 7 points, three studies (Döner and Taşcı 2022c; Oshvandi et al. 2021; Tsai et al. 2022) scored 6 points, two studies scored 5 points, and finally, one study (Azimpour et al. 2019) scored 4. All studies were of good quality as they had a quality score of 4 or more (Table 3).

**TABLE 3:** The evidence level and methodological qualities of studies.

Study	Was the research described as randomised?	Was the approach of randomisation appropriate?	Was the research described as blinding?	Was the approach of blinding appropriate?	Was there a presentation of withdrawals and dropouts?	Was there a presentation of the inclusion/exclusion criteria?	Was the approach used to assess adverse effects described?	Was the approach of statistical analysis described?	Total
Amrollahi et al. (2022)	1	1	1	1	1	1	1	1	8
Azimpour et al. (2019)	1	0	0	0	1	1	0	1	4
Döner and Taşcı (2022a)	1	1	0	0	1	1	1	1	6
Ghanbari et al. (2022)	1	1	0	0	1	1	0	1	5
Ghasemi et al. (2021)	1	1	1	1	1	1	1	1	8
Hashemi, Hajbagheri, and Aghajani (2015)	1	1	1	1	1	1	1	1	8
Ajorpaz et al. (2020)	1	1	1	1	1	1	0	1	7
Mohammadi et al. (2018)	1	1	1	1	1	1	1	1	8
Nasiri et al. (2019)	1	1	1	1	1	1	1	1	8
Oshvandi et al. (2021)	1	1	1	0	1	1	0	1	6
Shahgholian et al. (2016)	1	1	0	0	1	1	0	1	5
Tsai et al. (2022)	1	1	0	0	1	1	1	1	6

### 3.4 Risk of Bias Assessment

**FIGURE 2:**
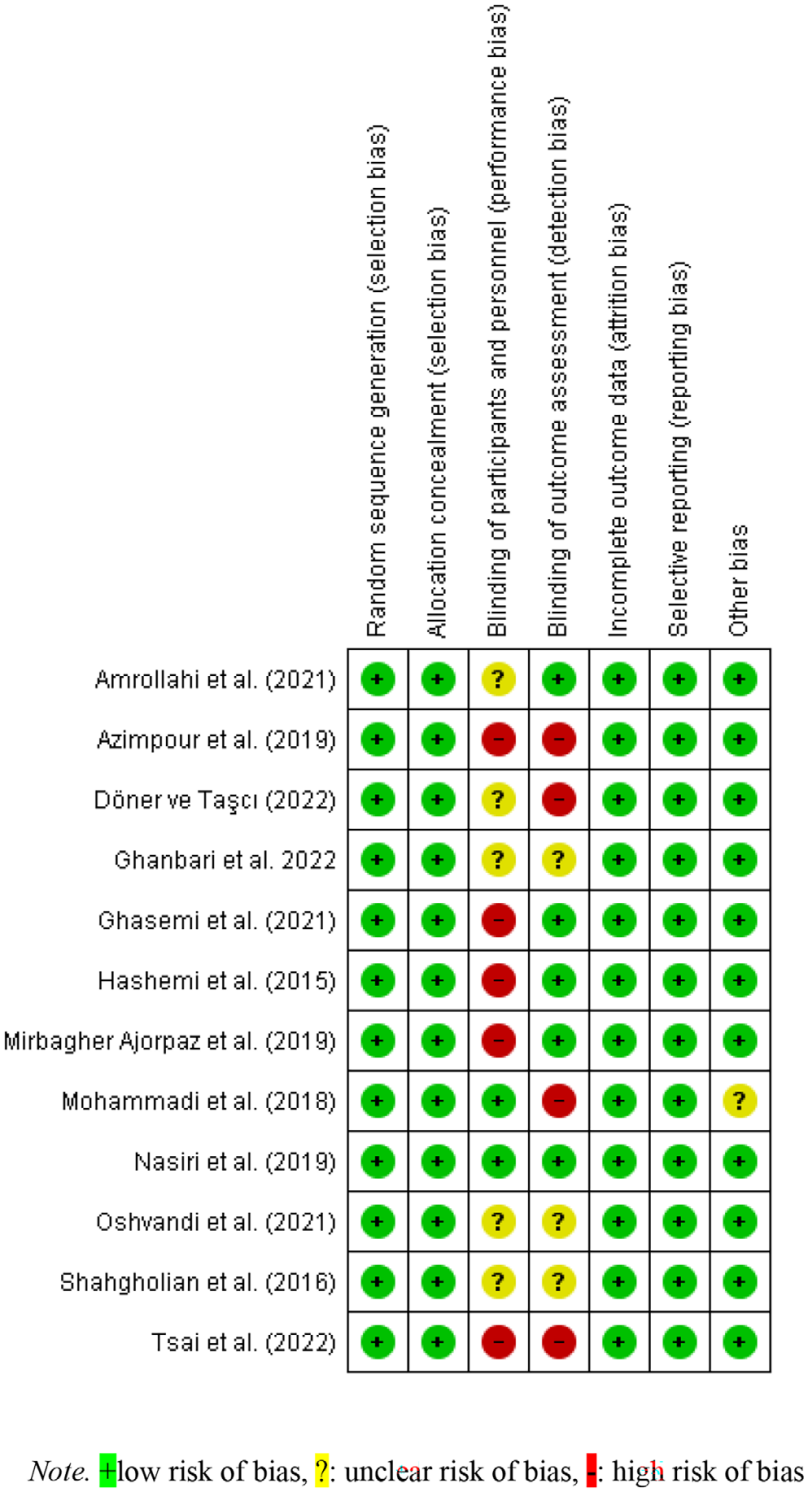
Risk of bias assessment for included studies.

**FIGURE 3:**
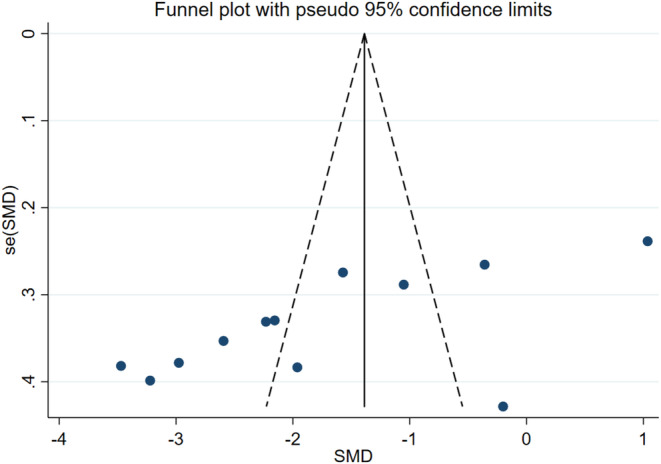
Funnel plot for publication bias.

All studies had low selection bias. The blinding process of the included studies was evaluated in terms of practitioners of interventions, participants, researchers and data analysts. When the blinding status of the participants was examined, two studies stated that the patients were blinded whether they were in the intervention or the control group. While five of the studies found insufficient information on whether participants were blinded, five of the studies stated that participants were not blinded due to the type of intervention. Considering the blinding status of the researchers who evaluated the outcome, four studies had a high bias due to not blinding and three studies had an uncertain bias because they did not provide information. The remaining five studies stated that the researchers who collected the data and performed the analysis were blinded and there was low bias. Data collection and reporting of data were sufficient in all included studies, so there was a low risk of reporting bias and attrition bias (Figure 2). The Egger regression test findings indicated the absence of publication bias among the included studies (*t* = −1.99, *p* = 0.061). Nevertheless, an asymmetry was observed in the funnel plot depicting these studies (Figure 3). This discrepancy underwent scrutiny via subgroup analysis and meta‐regression analysis.

### 3.5 Outcomes

**FIGURE 4:**
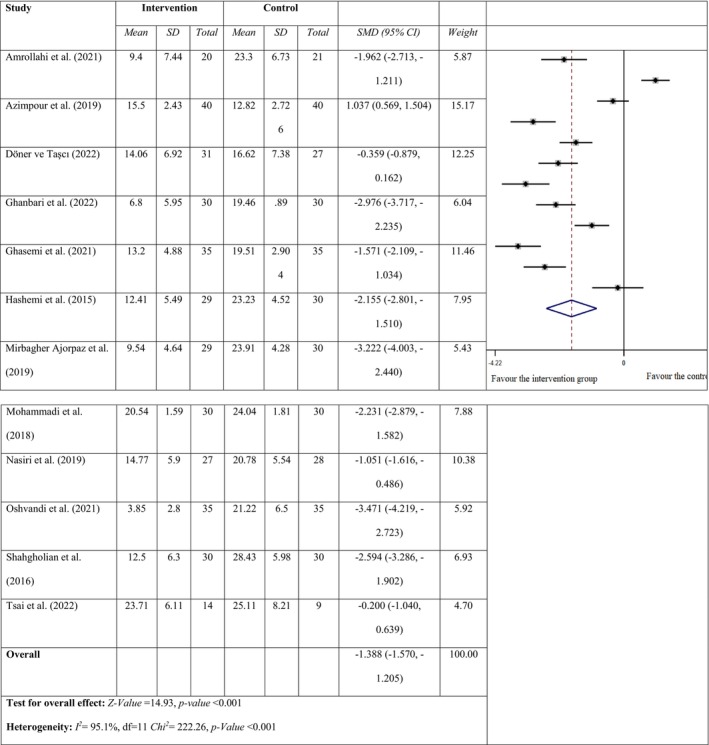
Forest plot for the RLS severity of the intervention and the control group.

**FIGURE 5:**
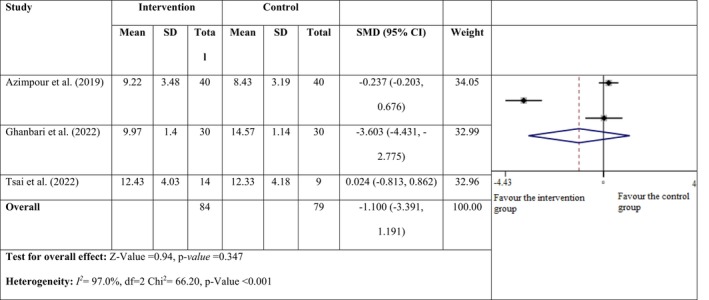
Forest plot for the sleep quality of the intervention and the control group.

The RLS severity of patients was evaluated with ‘The International RLS Study Group RLS rating scale for severity’. Assessing the impact of massage, reflexology and acupressure interventions on RLS severity in HD patients revealed significant heterogeneity among the 12 studies (*I* ^*2*^ = 95.1%; *p* < 0.001), prompting the adoption of a random effects model. Among these, nine studies indicated a notable reduction in RLS severity within the intervention group compared to the control group following massage and acupressure interventions. The combined results showed that massage and acupressure intervention provided a significant decrease in RLS severity in the intervention group compared to the control group (SMD = −1.388; 95% CI = −1.570, −1.205; *Z* = 14.93, *p* < 0.001; Figure 4). The SQ was assessed using the ‘Pittsburgh sleep quality index’ in only three of the studies (Azimpour et al. 2019; Ghanbari et al. 2022; Oshvandi et al. 2021). The random effects model was applied in this study because studies had a high degree of heterogeneity (*I* ^*2*^ = 97%; *p* < 0.001). The combined results revealed that massage and acupressure intervention had no effect on the SQ in the intervention group compared to the control group (SMD = −1.100; 95% CI = −3.391, 1.191; *Z* = 0.94, *p* = 0.347; Figure 5).

### 3.6 Subgroup Analysis

The type of intervention was divided into three subgroups: massage, reflexology and acupressure. In the subgroup analysis, it was determined that massage and acupressure reduced the severity of RLS as a result of the intervention, while reflexology had no effect. The HD treatment time of patients in the studies was divided into two subgroups less than 6 months and more than or equal to 6 months. There was no difference between the groups (*t* = −1.23, *p* = 0.246), and the severity of RLS was significantly reduced as a result of the intervention in both groups. Studies were divided into two according to whether or not to use oil in the intervention in the studies. As a result of the analysis, it was stated that the severity of RLS decreased as a result of the intervention in studies using oil. Considering the duration of the intervention in the studies, two subgroups were formed according to the duration of the intervention as subgroups less than 4 weeks and more than or equal to 4 weeks, and the severity of RLS was significantly reduced as a result of the intervention in both groups. Studies were divided into two subgroups according to the duration of the session, as less than 15 min and more than or equal to 15 min. In the analysis, RLS severity was significantly reduced as a result of the intervention in studies that intervened for more than 15 min per session (SMD = −2.288; 95% CI = ‐3.071, −1.504; *Z* = 5.72, *p* < 0.001), while studies that performed less than 15 min did not show any effect (SMD = −0.939; 95% CI = −2.174, 0.295; *Z* = 1.49, *p* = 0.136; Table 4).

**TABLE 4:** Subgroups analysis and meta‐regression of studies.

	Subgroups analysis							Meta‐regression			
	Subgroups	Number of studies	Std. mean difference	Lower limit	Upper limit	*Z* value	*p*	Coefficient	Std. err.	*t* value	*p*
Intervention	Massage	8	−1.575	−2.637	−0.513	2.91	**0.004**	−0.365	0.529	−0.69	0.505
	Reflexology	2	−1.595	−4.315	1.124	1.15	0.250				
	Acupressure	2	−2.400	−2.874	−1.927	9.94	**0.000**				
HD treatment time	< 6 month	7	−1.317	−2.523	−0.110	2.14	**0.032**	−0.966	0.783	−1.23	0.246
	≥ 6 month	5	−2.265	−3.148	−1.383	5.03	**0.000**				
Duration of intervention	< 4 week	3	−2.208	−3.557	−0.859	3.21	**0.001**	0.662	0.933	0.71	0.494
	≥ 4 week	9	−1.553	−2.565	−0.540	3.01	**0.003**				
Session duration	< 15 min	4	−1.090	−2.709	0.530	1.32	0.187	−1.352	0.719	−1.88	0.090
	≥ 15 min	8	−2.036	−2.904	−1.169	4.60	**0.000**				
Massage oil	Using	8	−2.071	−2.840	−1.302	5.28	**0.000**	1.092	0.809	1.35	0.207
	Not using	4	−0.992	−2.882	0.898	1.03	0.304				

*Note:* Bold values are statistically significant values *p* < 0.005.

Abbreviation: HD = haemodialysis.

## 4 Discussion

To the best of our knowledge, the current meta‐analysis is a recent meta‐analysis of the results of 12 studies investigating the effects of commonly used massage therapy, acupressure and reflexology practices on RLS symptom severity and SQ.

The current meta‐analysis showed that massage therapy and acupressure had a beneficial effect on RLS symptom severity in HD‐treated patients. In parallel with this finding, Xia et al. (2022) in their meta‐analysis study, reported that massage with or without herbal products reduced RLS symptoms and severity in HD patients. Chen et al. (2022) reported in a study that aromatherapy massage showed potential benefits in reducing the severity of RLS in HD patients. Huang et al. (2020) reported in a study, that acupuncture point stimulation reduced the severity of RLS in HD patients. In a systematic review study by Akbaş and Sözbir (2021), including individuals with primary and secondary RLS, it was determined that massage, acupuncture and near‐infrared light therapy applied to acupuncture points on the feet and legs reduced the severity of RLS. Huang et al. (2021), in a study comprising individuals with primary and secondary RLS, reported that acupuncture alone or in combination with other treatments was an effective treatment for RLS. In a systematic review study, Safarpour, Vaziri, and Jabbari (2023) reported that massage with lavender oil can improve RLS symptoms. In the meta‐analysis study by Kesik and Ersoy (2023), it was determined that aromatherapy massage has positive effects on RLS and muscle cramps. Massage therapy and acupressure interventions relax the muscles by solving muscle spasms, accelerate blood circulation, remove metabolites accumulated in the body and increase beta‐endorphin, serotonin and dopamine levels (Döner and Taşcı 2022b; Kesik and Ersoy 2023; Mehta et al. 2017). Based on the literature, massage therapy and acupressure may have physiological impacts on reducing HD patients' RLS symptoms and severity. The results of this study showed that massage therapy and acupressure interventions could be used to reduce RLS symptom severity in HD patients. The current meta‐analysis found no beneficial effect of reflexology on RLS severity. Chen et al. (2022) reported in a study that reflexology showed potential benefits in reducing the severity of RLS in patients treated for HD. Abbasi, Bastani, and Haghani (2018) determined that 10 min of foot reflexology massage with vaseline reduced RLS symptoms in elderly women with RLS. The beneficial effect of reflexology on RLS severity in the current meta‐analysis may be due to the small number of studies included in the meta‐analysis.

In aromatherapy, which is a sub‐branch of phytotherapy, it is emphasised that the intervention period should be at least 2–3 weeks for herbal extracts or products to take effect (Demirezer et al. 2021). Considering the intervention strategies, the results of this meta‐analysis showed that the duration of the intervention did not affect the change in RLS severity of HD patients as a result of the interventions. In the current meta‐analysis, the intervention time to the interventions varies between 3 and 8 weeks, and the intervention time is appropriate according to the literature. As there was no intervention period of less than 2–3 weeks, the results of the meta‐analysis came out in this way. In addition, most of the studies included in the current meta‐analysis did not specify a wash‐out period. It was stated in only one study that a 4‐week wash‐out period was given after the massage therapy (Azimpour et al. 2019). The presence of a residual effect is unclear, as most of the studies included in the current meta‐analysis did not have follow‐up evaluations. Future studies should provide evidence of any long‐term effects of these interventions.

Although the intervention time of classical massage varies regionally, the average lower extremity massage is 15–20 min (Döner and Taşcı 2022c). Considering the intervention strategies, this meta‐analysis showed that a session duration of 15 min or more in interventions reduced the severity of RLS, but did not affect the severity of RLS when less than 15 min of intervention were made per session. In the current meta‐analysis, the session duration of interventions varies in studies. In the study of Xia et al. (2022), it was reported that they could not perform a subgroup analysis because there was a limited number of studies to investigate the effect of different treatment durations on clinical outcomes. In the meta‐analysis study of Kesik and Ersoy (2023), it was determined that it could not be determined whether the differences in intervention strategies had a significant effect on the results. This finding in the current meta‐analysis highlights the importance of session duration in terms of the effect of interventions applied to HD patients with RLS in reducing the severity of RLS.

Considering intervention strategies, this meta‐analysis showed that the use of essential, base or lubricant oils in interventions reduced the severity of RLS in HD patients. The available meta‐analysis showed a beneficial effect on RLS symptom severity in studies that indicated the use of oil in interventions. In line with this finding, in the study of Xia et al. (2022), it was reported that massage with lavender oil improved RLS symptoms and reduced RLS severity in HD patients. Chen et al. combined massage with various oils as aromatherapy massage in a systematic review and meta‐analysis study. In the study, it was determined that aromatherapy massage reduced the severity of RLS. The study noted that due to limited evidence, it is difficult to distinguish whether these effects are the result of massage therapy or aromatherapy massage (Chen et al. 2022). Abbasi, Bastani, and Haghani (2018) determined that foot reflexology massage applied for 10 min with a neutral lotion (vaseline) improves the SQ in elderly women with RLS. It was determined that the majority of the studies included in the current meta‐analysis performed massage with lavender oil. Lavender oil reduces insomnia, sleep disorders and muscle pain (Babar et al. 2015). The reduction of RLS symptoms and severity in HD patients may be attributed to the physiological effects of massage and the effects of massage therapy oils.

The available meta‐analysis outcomes showed that interventions did not affect the SQ of HD patients. Chen et al. (2022) reported in a study that acupoint therapy can improve the SQ in HD patients. Huang et al. (2020) reported in a study that acupoint stimulation improves the SQ in HD patients. In a meta‐analysis study by Wang et al. (2020), it was determined that acupressure massage provided an improvement in the SQ in HD patients with sleep disorders. In a single‐blind RCT by Raissi et al. (2017), it was determined that acupuncture applied to patients with primary RLS improves the SQ.

## 5 Conclusion

Restless leg syndrome symptom management is very important for the treatment, and care of HD patients. The results of this meta‐analysis emphasise that massage therapy, and acupressure interventions applied to patients with RLS who receive HD treatment reduce the severity of RLS symptoms. Nurses contribute significantly to RLS symptom management by applying CIT methods that enable patients to recover, and strengthen in the treatment and care process with an individual and holistic perspective. In addition, studies with high methodological quality are needed to better evaluate the effects of massage therapy, acupressure and reflexology methods applied to HD patients with RLS on RLS symptom management, SQ and QoL. It is recommended to conduct RCTs for future studies that determine the effects of CIT methods on parameters such as RLS severity, daytime sleepiness, fatigue, QoL, depression and sleep in HD patients, including long‐term follow‐up, that has sufficient sample size, and that determine various intervention strategies (massage technique, total intervention time, follow‐up time, type and amount of oil used in massage therapy, development/non‐development of side effects against the oil used in interventions, at least 1 month wash‐out period, acupuncture points and number of stimulated points).

### 5.1 Strengths and Limitations

The current meta‐analysis has some limitations. First, the meta‐analysis does not include studies in languages other than English. Second, although different types of interventions with various contexts have been included, it is unclear whether this variability in approach and content significantly impacts outcomes. Third, unpublished studies are excluded in the current meta‐analysis, and it is, therefore unknown whether the results are affected by publication bias. On the other hand, the current meta‐analysis has strengths. First, the literature review has been carried out in the widest possible broad, and inclusive manner using several electronic databases. In addition, the methodological characteristics and biases of the studies included in the meta‐analysis are presented in detail. Second, it is the first meta‐analysis that determines intervention strategies (session duration and any oil use).

### 5.2 Implications for Practice

It is emphasised in the literature that RLS is common in HD patients, and this symptom reduces SQ and QoL of individuals. For this reason, it is very important to use CIT methods in the management of RLS symptoms in patients with RLS who receive HD treatment, to reduce the severity of RLS and improve the SQ and QoL in HD patients. This meta‐analysis provides evidence that massage therapy and acupressure have beneficial effects in reducing the severity of RLS in patients with RLS who are treated for HD. Nurses need to include relaxing and non‐invasive CIT methods such as massage therapy and acupressure in the care of HD patients to reduce the symptoms and severity of RLS experienced by individuals. Nurses should inform patients with RLS and their caregivers about the use of CIT methods in symptom management. In addition, it should be made routine to provide information and support to patients and caregivers about CIT methods in the management of RLS symptoms experienced by individuals in HD centres.

## Author Contributions

**Ayser Döner:** conceptualization, methodology, validation, formal analysis, investigation, resources, data curation, writing the original draft, reviewing and editing and visualization. **Sultan Taşci:** conceptualization, methodology, writing the original draft, reviewing and editing, visualization, supervision and project administration. **Aylin Bilgin:** methodology, validation, formal analysis, investigation, resources, data curation, writing the original draft, reviewing and editing and visualization.

## Ethics Statement

The authors have nothing to report.

## Conflicts of Interest

The authors declare no conflicts of interest.

## Supporting information


Data S1.

Data S2.

## Data Availability

The data that support the findings of this study are available on request from the corresponding author. The data are not publicly available due to privacy or ethical restrictions.
