# Supplementary material for: The Effect of Massage, Acupressure and Reflexology on Restless Legs Syndrome Severity and Sleep Quality in Patients Receiving Haemodialysis Treatment: A Systematic Review and Meta‐Analysis
Source: Nurs Open. 2025 Feb 4;12(2):e70135. doi: 10.1002/nop2.70135 (PMC11794833; doi:10.1002/nop2.70135)
Supplement: Supplementary file 2 — Data S2. [file NOP2-12-e70135-s002.docx]

**Supplementary material 2.** Search strategy for each database.

| PubMed | Title/Abstract= “Restless leg syndrome” OR “Restless Legs Syndrome” OR “Willis-Ekbom disease” OR “RLS” OR “WED” OR “uremic RLS” OR “secondary RLS")  AND  Title/Abstract= (“Hemodialysis”)  AND  Title/Abstract= “Aromatherapy” OR “Aroma” OR “Massage” OR “Reflexology” OR “Acupressure” OR “Acupuncture” OR “Acupoint”) |
| --- | --- |
| CENTRAL | \| #1 \| MeSH descriptor: [Restless leg syndrome] explode all trees \| \| --- \| --- \| \| #2 \| MeSH descriptor: [Restless Legs Syndrome] explode all trees \| \| #3 \| MeSH descriptor: [Willis-Ekbom disease] explode all trees \| \| #4  #5 \| MeSH descriptor: [RLS] explode all trees  MeSH descriptor: [WED] explode all trees \| \| #6 \| MeSH descriptor: [uremic RLS] explode all trees \| \| #7 \| MeSH descriptor: [secondary RLS] explode all trees \| \| #8 \| #1 OR #2 OR #3 OR #4 OR #5 OR #6 OR #7 \| \| #9 \| MeSH descriptor: [Hemodialysis] explode all trees \| \| #10 \| MeSH descriptor: [Aromatherapy] explode all trees \| \| #11 \| MeSH descriptor: [Aroma] explode all trees \| \| #12 \| MeSH descriptor: [Massage] explode all trees \| \| #13 \| MeSH descriptor: [Reflexology] explode all trees \| \| #14 \| MeSH descriptor: [Acupressure] explode all trees \| \| #15 \| MeSH descriptor: [Acupoint] explode all trees \| \| #16 \| MeSH descriptor: [Acupuncture] explode all trees \| \| #17 \| #10 OR #11 OR #12 OR #13 OR #14 OR #15 OR #16 \| \| #18 \| #8 AND #9 AND #17 \| |
| EBSCO | Title/Abstract= “Restless leg syndrome” OR “Restless Legs Syndrome” OR “Willis-Ekbom disease” OR “RLS” OR “WED” OR “uremic RLS” OR “secondary RLS")  AND  Title/Abstract= (“Hemodialysis”)  AND  Title/Abstract= “Aromatherapy” OR “Aroma” OR “Massage” OR “Reflexology” OR “Acupressure” OR “Acupuncture” OR “Acupoint”) |
| ScienceDirect | (“Restless leg syndrome” OR “Restless Legs Syndrome” OR “Willis-Ekbom disease” OR “RLS” OR “WED” OR “uremic RLS” OR “secondary RLS")  AND  (“Hemodialysis”)  AND  (“Aromatherapy” OR “Aroma” OR “Massage” OR “Reflexology” OR “Acupressure” OR “Acupuncture” OR “Acupoint”) |
| Web of Science | TS(Topic)= (“Restless leg syndrome” OR “Restless Legs Syndrome” OR “Willis-Ekbom disease” OR “RLS” OR “WED” OR “uremic RLS” OR “secondary RLS")  AND  TS(Topic)= (“Hemodialysis”)  AND  TS(Topic)= (“Aromatherapy” OR “Aroma” OR “Massage” OR “Reflexology” OR “Acupressure” OR “Acupuncture” OR “Acupoint”) |
